# Integrative Taxonomy Reveals a Candidate Lineage Within the *Rhinolophus macrotis* Group

**DOI:** 10.3390/biology15110846

**Published:** 2026-05-28

**Authors:** Jinhua Cong, Jiajun Zhang, Haoran Yu, Jinhong Lei, Guiyin Miao, Heran Yang, Qiuchen Li, Zhejia Zhang, Gábor Csorba, Keping Sun, Tong Liu

**Affiliations:** 1 Jilin Provincial International Cooperation Key Laboratory for Biological Control of Agricultural Pests, Jilin Agricultural University, Changchun 130118, China; 2 Jilin Provincial Key Laboratory of Animal Resource and Ecological Security, Jilin Agricultural University, Changchun 130118, China; 3 Department of Zoology, Hungarian Natural History Museum, 1088 Budapest, Hungary; 4 Key Laboratory of Vegetation Ecology of Education Ministry, Institute of Grassland Science, Northeast Normal University, Changchun 130024, China

**Keywords:** integrative taxonomy, phenotype, mitogenome, multi-locus, bat

## Abstract

Accurate species delimitation is fundamental yet challenging, particularly in recently diverged, phenotypically conservative taxa such as bats. The “*Rhinolophus macrotis* group” represents one of the most taxonomically contentious groups among horseshoe bats. During field surveys in Southwest China, we discovered an unidentified *Rhinolophus* sp. occurring sympatrically with *R. osgoodi* and *R. episcopus*, sharing broad morphological affinities with recognized species of the “*R. macrotis* group.” To explore its taxonomic status, we employed an integrative approach combining morphological, acoustic, and multi-locus genetic (mitogenomic and nuclear) data. Phenotypically, *Rhinolophus* sp. closely resembles *R. osgoodi* but can be distinguished by its divergent echolocation resting frequency. Genetically, while mitochondrial data deeply nested *Rhinolophus* sp. within *R. osgoodi* with a shallow divergence time, phylogenies based on two nuclear introns positioned it closer to two other species, *R. episcopus* and *R. siamensis*. Species delimitation based on these genetic markers revealed a pattern of mitochondrial subdivision contrasted by overly conservative nuclear signals. Such mito-nuclear discordance suggests a complex evolutionary history that complicates taxonomic assignments. Given that only three specimens of *Rhinolophus* sp. were available, which precludes a robust assessment of intraspecific variation, we provisionally designate it as a candidate lineage within the “*R. macrotis* group,” warranting future validation with additional comprehensive evidence. This study highlights the indispensable utility of integrative taxonomy in uncovering hidden diversity and provides insights into chiropteran evolutionary history.

## Simple Summary

Accurate species delimitation in recently diverged, phenotypically conservative taxa remains a challenge. The *Rhinolophus macrotis* group is highly contentious among horseshoe bats. During field surveys in Southwest China, we discovered an unidentified *Rhinolophus* sp., which can be distinguished from its closely related congeners by its divergent echolocation frequency. Furthermore, mitochondrial phylogenies deeply nested *Rhinolophus* sp. within *R. osgoodi* with a recent divergence, whereas nuclear data placed it closer to two other species, *R. episcopus* and *R. siamensis*. Given that only three specimens of *Rhinolophus* sp. were available, we provisionally designate it as a candidate lineage within the *R. macrotis* group, warranting future genomic validation. This study highlights the indispensable utility of integrative taxonomy in uncovering hidden chiropteran diversity.

## 1. Introduction

Elucidating the genetic mechanisms and processes underlying speciation has long been a central goal in evolutionary biology [1,2]. Accurate species delimitation is critical to this objective, yet it remains challenging across numerous cryptic taxa [3,4]. Typically, genetically independent lineages exhibit phenotypic divergence, yielding congruent delimitation results. However, phenomena such as phenotypic plasticity and morphological stasis can result in highly similar morphologies among independently evolving lineages, leading to the formation of cryptic or sibling species [5,6,7]. Furthermore, natural selection, incomplete lineage sorting, and asymmetrical introgression can lead to discordant delimitation outcomes across genes with different inheritance patterns [8,9]. Consequently, phenotype-genotype discordance and mito-nuclear discordance pose substantial challenges to species delimitation and phylogenetic reconstruction, particularly for understudied non-model organisms.

The application of integrative taxonomy provides a robust framework to address these challenges [10,11]. By integrating diverse lines of evidence such as genetic, morphological, ecological, and geographical data, this approach enhances the detection of discordances and increases the accuracy of species delimitation [12,13,14,15]. Because such discordances often imply unique evolutionary processes, such as convergent evolution or interspecific hybridization [16,17], integrative taxonomy also presents new opportunities for evolutionary research [18,19]. While its application among morphologically conservative taxa has increased over the past decade [20,21,22,23,24,25], its overall utilization remains disproportionately limited relative to undiscovered biodiversity.

Chiroptera represents the second most diverse order of mammals, comprising 1500 species and accounting for approximately 20% of all mammalian diversity [26]. Within this order, the family Rhinolophidae has undergone rapid adaptive radiation over the past five million years [27,28], giving rise to numerous recently diverged taxa and cryptic species complexes [29,30]. High levels of morphological stasis among these closely related species, compounded by the cryptic nature of their nocturnal ecology, have resulted in numerous unresolved taxonomic issues and an underestimation of Rhinolophidae diversity [29].

The “*R. macrotis* group” stands out as one of the most taxonomically contentious groups within horseshoe bats, with a complex history of cryptic species discovery and reclassification [31,32,33,34,35,36,37,38,39,40]. Originally described from Nepal in 1844, *R. macrotis* is a medium-sized horseshoe bat [41]. Following this, several new taxa belonging to the *macrotis* group were reported in China, including *R. episcopus*, *R. e. caldwelli*, and *R. rex* [39]. Subsequent taxonomic revisions classified *R. episcopus* and *R. e. caldwelli* as subspecies of *R. macrotis* [42]. As an increasing number of taxa were subsumed under *R. macrotis*, it came to be regarded as a single species comprising numerous subspecies and widely distributed across South and Southeast Asia [43,44,45]. Over the past two decades, investigations across China and Vietnam have revealed that the nominal *R. macrotis* distributed in these regions actually represents a species complex comprising multiple distinct taxa [31,34,35,36,37,38]. Building upon this, Zhang et al. (2018) recovered a highly supported monophyletic clade and formally designated the “*R. macrotis* group”, which initially comprised the *R. macrotis* complex, *R. rex*, *R. marshalli*, and *R. paradoxolophus* [32]. To systematically resolve taxonomic controversies within the *R. macrotis* complex, Liu et al. (2019) integrated multiple lines of evidence and delimited the complex into three distinct species: *R. episcopus* (including *R. e. episcopus*, *R. e. caldwelli*, and *R. episcopus* ssp.), *R. siamensis*, and *R. osgoodi* [33]. Most recently, Tu et al. (2023) revised the taxonomic status of *R. rex* and its close relatives, *R. paradoxolophus* and *R. schnitzleri*, synonymizing them into a single species [46]. As a result of these successive taxonomic revisions, the “*R. macrotis* group” in mainland Southeast Asia currently comprises five recognized species: *R. episcopus*, *R. siamensis*, *R. osgoodi*, *R. rex*, and *R. marshalli*.

**Figure 1:**
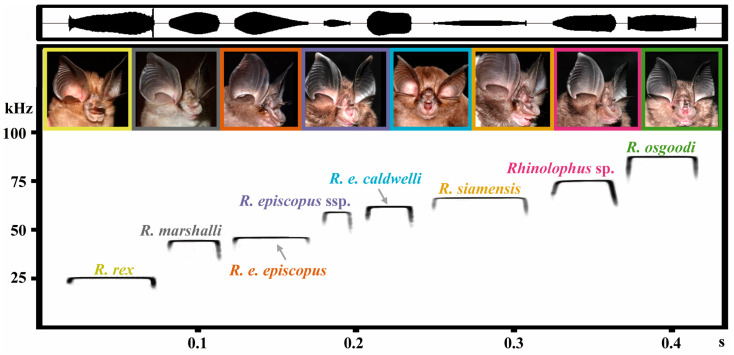
Morphological and acoustic characteristics of *Rhinolophus* sp. and recognized taxa within the “*Rhinolophus macrotis* group.” Distinct colors correspond to different species or subspecies. Photographs of *R. rex* and *R. marshalli* are reproduced with permission from Tu et al. (2017) [31], John Wiley and Sons. Photographs of *R. episcopus*, *R. siamensis*, and *R. osgoodi* are reproduced with permission from Liu et al. (2019) [33], Elsevier. The photograph of the *Rhinolophus* sp. was taken during our field surveys. The specific specimen vouchers for the pictured individuals, ordered from left to right, are as follows: *R. rex* (NH2016-66), *R. marshalli* (IEBR.VN14-0212), *R. e. episcopus* (HUN1636), *R. episcopus* ssp. (YN07300), *R. e. caldwelli* (FJ1621), *R. siamensis* (YN07148), *Rhinolophus* sp. (YN16102), and *R. osgoodi* (YN16158).

During field surveys conducted in Yunnan province, China, between 2016 and 2017, we collected three specimens of an unidentified horseshoe bat (*Rhinolophus* sp.). While its overall phenotype aligns with the species in “*R. macrotis* group”, its echolocation call frequency differs distinctly from all other members of this group (Figure 1). Moreover, the noseleaf of *Rhinolophus* sp. displays an intermediate morphology relative to its two sympatric species, *R. episcopus* ssp. and *R. osgoodi*. Specifically, its lancet shape falls between the broadly rounded structure of *R. episcopus* and the halberd-like form of *R. osgoodi*. Additionally, the secondary leaflet is moderately developed, distinguishing it from both the prominent leaflet of *R. episcopus* and the completely absent leaflet in *R. osgoodi*. These overall morphological affinities, coupled with distinct acoustic and noseleaf traits, suggest that *Rhinolophus* sp. may represent a novel cryptic species, necessitating comprehensive morphological and genetic data to confirm its taxonomic status.

In this study, we combine morphological, acoustic, and genetic data to conduct an integrative taxonomic and phylogenetic assessment of *Rhinolophus* sp. and its close relatives within the “*R. macrotis* group.” Our primary objectives were to: (1) quantify the degree of phenotypic divergence between *Rhinolophus* sp. and its congeners; (2) assess the extent of their genetic differentiation; and (3) elucidate the taxonomic status and phylogenetic relationships within the group. This study serves as an empirical model for integrative taxonomic practice in bats. Our findings offer insights into complex evolutionary histories, advancing our comprehension of speciation processes and cryptic diversity within Chiroptera.

## 2. Materials and Methods

### 2.1. Samples Collection

**Figure 2:**
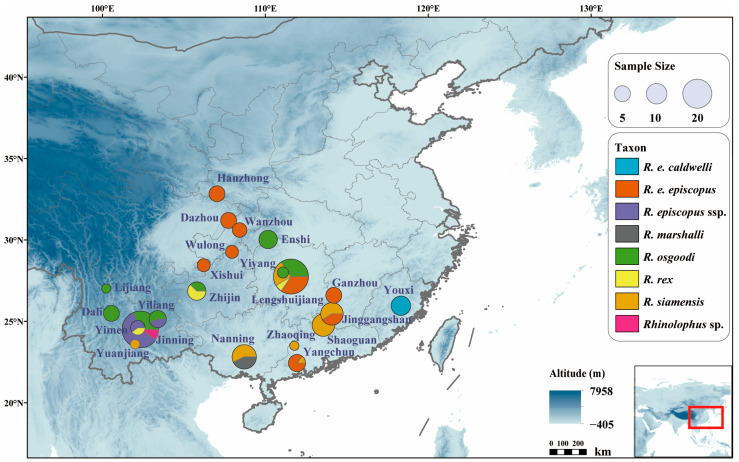
Sampling locations of *Rhinolophus* sp. and recognized taxa within the “*Rhinolophus macrotis* group.” Pie charts indicate the taxonomic composition at each sampling site, with colors corresponding to distinct taxa, and chart sizes scaled proportionally to the sample size at that locality. The inset map shows the location of the study region within Asia, highlighted by a red bounding box. Detailed sample information is available in Supplementary Table S1.

Between 2006 and 2024, a total of 189 specimens were captured using mist-nets across southern China, comprising 3 individuals of *Rhinolophus* sp. and 186 individuals of closely related taxa (Figure 2, Table S1). To minimize the impact on local populations, all sampled individuals were confirmed to be adults in a non-reproductive state (i.e., non-pregnant and non-lactating). Following phenotypic measurement and acoustic recordings in a temporary field laboratory, wing membrane biopsies were obtained from each individual using 3 mm sterile biopsy punches and preserved in absolute ethanol. After confirming their health status, the bats were released at the original capture sites. In cases of accidental mortality, muscle tissues were sampled, and skulls were extracted following Bates et al. (2005) [47]. The wet-preserved voucher specimens were deposited at Northeast Normal University (NENU) and Jilin Agricultural University (JLAU), with detailed information provided in Table S2. Additionally, specimen YN16102 is housed at the Hungarian Natural History Museum (HNHM) under voucher number CSOCH 102.

### 2.2. Morphological and Acoustic Data Acquisition

Seven external morphological parameters were measured using digital calipers (accurate to 0.01 mm): forearm length (FA), ear length (EL), ear width (EW), sella height (SH), horseshoe width (HW), tail length (TAIL), and hindfoot length (HF). For *Rhinolophus* sp., these measurements were restricted to two individuals to preclude inaccuracies caused by wet preservation. Eleven craniodental parameters were measured to the nearest 0.01 mm, including greatest length of skull (GLS), condylocanine length (CCL), braincase height (BCH), braincase breadth (BB), interorbital breadth (IOB), zygomatic breadth (ZB), breadth across upper canines (C^1^–C^1^), outer breadth across upper canine (C^1^C^1^), upper canine–third molar length (C–M^3^), breadth across third upper molars (M^3^–M^3^), and mastoid breadth (MB). Due to cranial damage in one specimen and the necessity to preserve the morphological integrity of another as a wet-preserved voucher, cranial extraction and measurements for *Rhinolophus* sp. were limited to a single specimen.

Echolocation calls of *Rhinolophus* sp. and its congeners in good physical condition were recorded at ambient summer temperatures (28–30 °C) using an Avisoft UltraSoundGate system (Avisoft Bioacoustics, Glienicke/Nordbahn, Germany). To minimize frequency variations associated with Doppler-shift compensation (DSC) during flight, calls were recorded from single individuals hanging freely inside a light-mesh bag. The microphone was positioned 50 cm from the bat’s head. Recordings were initiated 30 s after the bat was placed in the bag, with each recording session lasting for at least three minutes per individual. Acoustic signals were digitized at a sampling rate of 375 kHz with 16-bit resolution. For each individual, ten high-quality pulses were selected to measure the resting frequency (RF; defined as the frequency of maximal energy) using Avisoft-SASLab Pro v5.3.2, with analytical parameters following those described by Liu et al. (2021) [48]. The mean RF value was then calculated for each individual.

All individual data, including external morphological measurements and resting frequencies, are listed in Table S1, while craniodental parameters are summarized in Table S2. Given that previous studies have demonstrated a lack of significant sexual dimorphism in these taxa [33,46], phenotypic data from both sexes were combined for all subsequent analyses.

### 2.3. Genetic Data Acquisition

Genomic DNA was extracted from freshly acquired wing membranes or muscle tissues using UNIQ-10 column animal genomic DNA isolation kits (Sangon Biotech, Shanghai, China). DNA concentration and purity were assessed using a NanoDrop microvolume spectrophotometer (Thermo Fisher Scientific, Waltham, MA, USA). The mitochondrial *Cytb* gene and two nuclear introns (*Acox2* and *Chd1*) were amplified following the primers and protocols described by Sun et al. (2016) [34] and Zhang et al. (2018) [32].

Furthermore, to comprehensively resolve the phylogenetic relationships of *Rhinolophus* sp. from a mitogenomic perspective, specific primers were designed based on the publicly available reference mitogenomes from the “*R. macrotis* group” using NCBI Primer-BLAST v2.5.0 [49]. Using these primers, we amplified and sequenced the complete mitochondrial genomes of three *Rhinolophus* sp. individuals, one *R. osgoodi*, and one *R. e. caldwelli* via Sanger sequencing. Detailed primer sequences and optimized PCR conditions are provided in Table S3.

Sequence assembly, alignment, and annotation were performed in Geneious v8.1.8 (https://www.geneious.com). For the 13 protein-coding genes (PCGs), annotations were further verified by translating nucleotide sequences into amino acids to ensure the absence of premature stop codons. With the addition of these newly generated sequences, complete mitogenomic data for all recognized species and subspecies within the “*R. macrotis* group” have been successfully compiled.

The newly generated nucleotide sequences in this study have been deposited in GenBase [50] at the National Genomics Data Center, China National Center for Bioinformation [51], under accession numbers C_AA165316 to C_AA165355 and C_AA166916 to C_AA166920. A comprehensive list of all newly generated sequences, along with the publicly available genetic data, is provided in Tables S4 and S5.

### 2.4. Integrative Taxonomic Analyses

To preliminarily assess morphological divergence, principal component analysis (PCA) was performed independently on the external and craniodental datasets, with scatter plots generated based on the first three principal components. Subsequently, morphological clustering and taxonomic unit identification were conducted using Gaussian mixture models (GMMs) implemented in the R package mclust v6.1.2 [52]. Given the highly unbalanced sample sizes, phenotypic differentiation between *Rhinolophus* sp. and each of its closely related taxa was further evaluated through detailed comparisons of resting frequency and major morphological parameters selected based on their highest PCA loadings (Table S6). Because the datasets deviated from a normal distribution, non-parametric two-sample Wilcoxon rank-sum tests were employed for all pairwise comparisons, and box plots were generated to visualize the data distribution and statistical differences. Due to the availability of only a single skull specimen for *Rhinolophus* sp., these statistical tests were restricted to external morphology and acoustic data. All statistical analyses and visualizations were performed in R v4.5.0 [53].

The optimal nucleotide substitution models for the *Cytb* gene and the concatenated sequences of the two nuclear introns were determined separately using ModelFinder [54] implemented in IQ-TREE v2.4.0 [55]. Detailed information regarding the selected best-fit models for all datasets is provided in Table S7. Phylogenetic relationships were reconstructed using maximum likelihood (ML) and Bayesian inference (BI) methods. The ML analysis was implemented in IQ-TREE with 1000 ultrafast bootstrap replicates. The BI analysis was run in MrBayes v3.1 [56] for 5,000,000 generations, with the initial 25% of sampled trees discarded as burn-in. To assess species boundaries, candidate taxonomic units were delineated based on the mitochondrial and nuclear datasets using uncorrected *p*-distances via the ABGD web-server at https://spartexplorer.mnhn.fr/delimitation (accessed on 20 December 2025). Furthermore, we performed tree-based species delimitation using the multi-rate Poisson tree processes (mPTP) algorithm [57]. The multi-rate coalescent model was applied to the inferred ML trees, and the Markov Chain Monte Carlo (MCMC) analysis was run for 50,000,000 generations, with sampling every 5000 generations and the initial 10% discarded as burn-in. To explicitly quantify species-level divergence and facilitate comparisons with established mammalian thresholds, interspecific genetic distances for the *Cytb* gene were calculated in MEGA v11 [58] using the Kimura 2-parameter (K2P) model.

### 2.5. Mitogenomic Phylogeny Reconstruction and Divergence Time Estimation

While the *Cytb* and nuclear datasets provided extensive taxonomic sampling for species delimitation, we further utilized the complete mitochondrial genomes and the concatenated sequences of the 13 PCGs to reconstruct mitogenomic phylogenetic relationships within the “*R. macrotis* group” and estimate the time to the most recent common ancestor (TMRCA). The BI and ML analyses based on the entire mitochondrial genomes and concatenated sequences of the 13 PCGs were conducted following the aforementioned protocols. In all phylogenetic analyses, *R. pusillus* was designated as the outgroup.

Subsequently, the TMRCA was estimated using BEAST v1.8.2 [59] based on the concatenated 13 PCGs dataset. Following the previous study [60], the mitochondrial substitution rate was set to 0.013 substitutions per site per million years. The MCMC chain was set to 20,000,000 generations, sampling every 1000 generations. Convergence of the MCMC chains was assessed in Tracer v1.7 [61], ensuring that all effective sample size (ESS) values exceeded 200. A maximum clade credibility tree was then summarized using TreeAnnotator v1.10, with the first 25% of sampled trees discarded as burn-in. All resulting phylogenetic topologies and the BEAST-derived chronogram were visualized in FigTree v1.4.4.

## 3. Results

### 3.1. Multivariate Morphometric Differentiation

**Figure 3:**
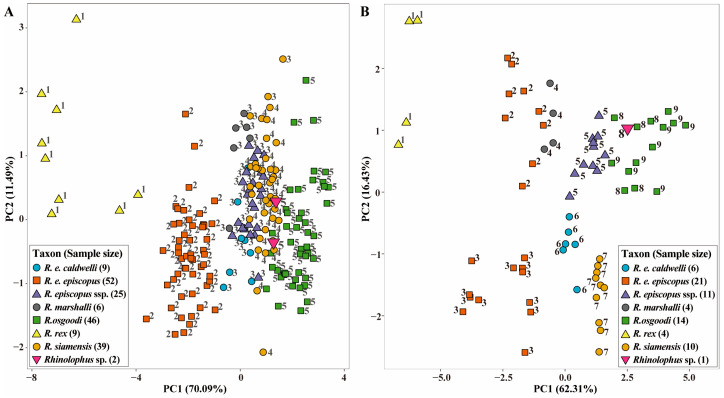
Multivariate morphometric analyses of *Rhinolophus* sp. and its allied taxa. Principal component analysis (PCA) scatter plots and Gaussian mixture model (GMM) clustering results based on (**A**) seven external morphological parameters (yielding five clusters) and (**B**) 11 craniodental morphological parameters (yielding nine clusters). Different colors and geometric shapes denote a priori species or subspecies identifications, with numbers in parentheses indicating the corresponding sample sizes for each taxon. Numerical labels adjacent to the data points represent their objective cluster assignments derived from the GMM analysis. Detailed cluster assignments for individual specimens are provided in Tables S1 and S2.

Through the integration of newly acquired and previously published data, we compiled comprehensive morphological and acoustic records for *Rhinolophus* sp. and its allied taxa within the “*R. macrotis* group.” Our finalized datasets incorporated external morphological measurements for 188 individuals, craniodental measurements for 71 individuals, and acoustic data for 186 individuals (Tables S1 and S2). Across both external and craniodental datasets, the morphological separation revealed by PCA was highly congruent with the clustering identified via Gaussian mixture modeling (Figure 3).

Based on the seven external morphological parameters, the first three principal components (PC1, PC2, and PC3) accounted for 87.49% of the cumulative variance (Figure 3A and Figure S1A). Gaussian mixture modeling identified five robust clusters (Figure S2A). The two individuals of *Rhinolophus* sp. were assigned to Cluster 5, exhibiting the greatest morphological affinity with *R. osgoodi* (which was predominantly classified into Clusters 4 and 5). Despite this affinity, *Rhinolophus* sp. remained generally distinct from other *Rhinolophus* taxa, particularly the large-sized *R. rex* and *R. e. episcopus*.

Similar results were obtained from the analysis of the 11 craniodental parameters, with the first three PCs explaining 87.97% of the total variance (Figure 3B and Figure S1B). GMM clustering identified nine clusters (Figure S2B), with the single individual of *Rhinolophus* sp. grouping into Cluster 8. Sharing this cluster exclusively with *R. osgoodi* (which occupied Clusters 8 and 9), *Rhinolophus* sp. exhibited the highest cranial affinity to *R. osgoodi* across the first three PC axes.

**Figure 4:**
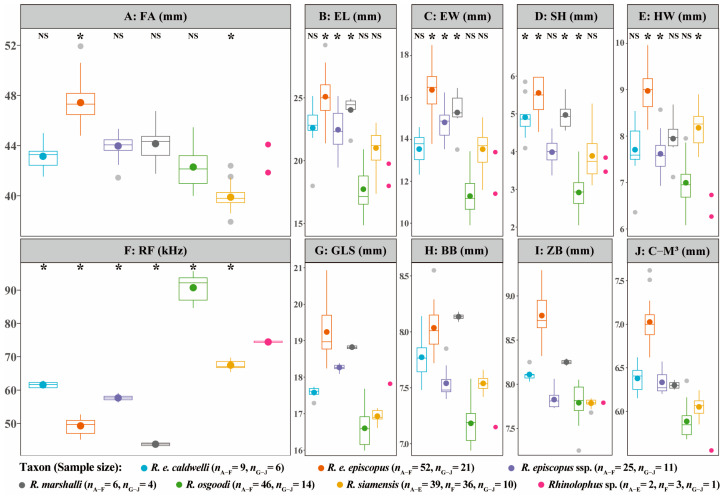
Comparisons of phenotypic and acoustic traits between *Rhinolophus* sp. and its allied taxa. Box plots illustrate variations in external morphological parameters (**A**–**E**), echolocation resting frequencies (**F**), and craniodental morphological parameters (**G**–**J**). Raw data points rather than box plots are presented for taxa with restricted sample sizes (*n* < 3). The light grey dots represent outlier data points. Asterisks (*) denote statistically significant differences compared to *Rhinolophus* sp. (Wilcoxon rank-sum tests, *p* < 0.05), while “NS” indicates no significant difference. Statistical tests were not performed on craniodental traits due to the limited sample size of *Rhinolophus* sp. Different colors represent distinct taxa, with sample sizes for each characteristic category detailed in the bottom legend. Abbreviations: FA, forearm length; EL, ear length; EW, ear width; SH, sella height; HW, horseshoe width; RF, resting frequency; GLS, greatest length of skull; BB, braincase breadth; ZB, zygomatic breadth; C–M^3^, upper canine–third molar length.

To ensure the accuracy of fine-scale phenotypic assessments, the morphologically highly divergent *R. rex* was excluded from further pairwise comparisons. The results demonstrated that while the overall body size of *Rhinolophus* sp. is comparable to that of *R. e. caldwelli*, *R. episcopus* ssp., *R. marshalli*, and *R. osgoodi* (Figure 4A), it exhibits statistically significant differences in parameters associated with outer ear and noseleaf traits (Figure 4B–E). Cranially, the single specimen of *Rhinolophus* sp. exhibits a relatively shorter C–M^3^ (Figure 4J), though potential intraspecific variation cannot be excluded. Furthermore, acoustic analyses revealed that the resting frequency (RF) of *Rhinolophus* sp. (74.43 ± 0.46 kHz, *n* = 3) is distinct from those of its close relatives (Figure 4F).

### 3.2. Interspecific Genetic Divergence

**Figure 5:**
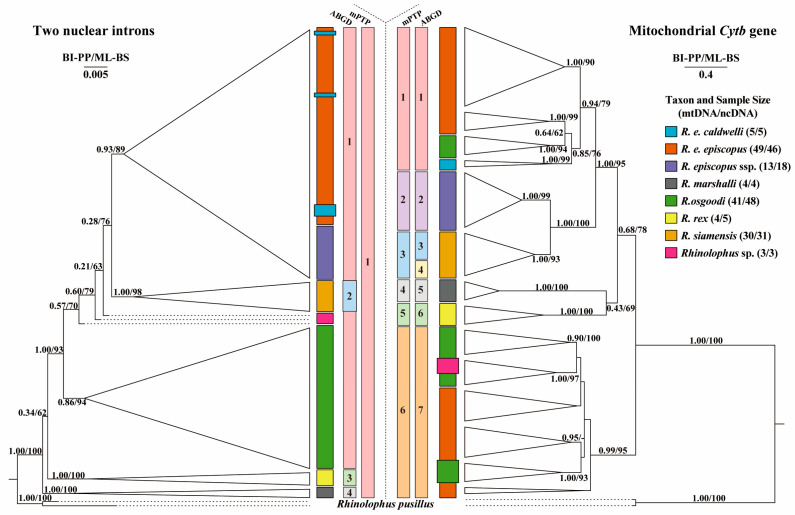
Phylogenetic relationships and species delimitation results based on two concatenated nuclear introns (**left**) and the mitochondrial *Cytb* gene (**right**). Solid-colored blocks at the tree tips denote a priori taxonomic identifications of *Rhinolophus* sp. and its allied taxa, with colors corresponding to the species legend. Numbers in parentheses within the legend indicate the sample sizes for the mitochondrial and nuclear datasets, respectively. The adjacent numbered vertical bars indicate putative species delineated by the ABGD and mPTP analyses. Numbers at the nodes indicate Bayesian posterior probabilities (BI-PP) and maximum-likelihood bootstrap support (ML-BS) values, respectively. *Rhinolophus pusillus* was used as the outgroup.

By integrating previously published and newly sequenced genetic data, we obtained 163 concatenated nuclear intron sequences and 151 mitochondrial *Cytb* gene sequences for *Rhinolophus* sp. and its close relatives. These datasets yielded 116 and 80 unique haplotypes for phylogenetic reconstruction, respectively. While topologies derived from BI and ML analyses were highly congruent within each dataset, substantial mito-nuclear discordance was observed regarding both phylogenetic relationships and species delimitation (Figure 5).

Overall, the mitochondrial gene revealed more extensive intraspecific genetic differentiation and interspecific admixture compared to the nuclear markers (Figure 5). In the nuclear phylogeny, *Rhinolophus* sp. exhibited the closest phylogenetic affinities to the three subspecies of *R. episcopus*, albeit with weak nodal support (BI-PP = 0.57; ML-BS = 70%). Furthermore, all *R. osgoodi* samples formed a distinct monophyletic clade, which was recovered as sister to the aforementioned clade (BI-PP = 1.00; ML-BS = 93%). Conversely, the mitochondrial topology resolved *Rhinolophus* sp. as nested within a subset of *R. osgoodi*. This mixed subclade subsequently clustered with the remaining *R. osgoodi* and some *R. e. episcopus* individuals to form a broader, well-supported monophyletic clade (BI-PP = 0.99; ML-BS = 95%).

Species delimitation via ABGD based on the nuclear and mitochondrial *Cytb* datasets identified four and seven putative species, respectively (Figure 5). Broadly congruent with the nuclear phylogeny, the four nuclear-derived putative species corresponded to previously recognized taxonomic boundaries, with the exception that *Rhinolophus* sp., *R. episcopus*, and *R. osgoodi* were lumped into a single putative species. In contrast, the mitochondrial ABGD analysis yielded a greater number of fine-scale taxonomic partitions, successfully delimiting *R. episcopus* ssp., *R. siamensis*, *R. rex*, and *R. marshalli* as distinct taxonomic entities. Consistent with the pronounced intraspecific genetic divergence observed in the mitochondrial topology, the ABGD results split individuals of *R. e. episcopus* and *R. osgoodi* across two putative species, while merging *Rhinolophus* sp. with specific individuals from both *R. e. episcopus* and *R. osgoodi* into a single composite putative entity.

Subsequent tree-based species delimitation using the mPTP algorithm yielded more conservative estimates across both datasets (Figure 5). For the nuclear dataset, the analysis conservatively collapsed all focal individuals into a single putative taxonomic entity. In contrast, the multi-rate coalescent model applied to the mitochondrial *Cytb* dataset delineated six putative species. These mitochondrial results largely mirrored the ABGD partitions, differing only in the further lumping of all *R. siamensis* individuals.

Estimates of K2P genetic distances derived from the mitochondrial *Cytb* dataset further corroborated these complex phylogenetic patterns (Table 1). The minimum interspecific sequence divergence was recorded between *Rhinolophus* sp. and *R. osgoodi* (1.4–2.0%), a range slightly lower than the divergence observed between *R. osgoodi* and *R. e. episcopus* (1.7–2.3%), as well as between *Rhinolophus* sp. and *R. e. episcopus* (2.2–3.0%). Moreover, excluding these closely related lineages, the genetic distances between *Rhinolophus* sp. and all other taxa (including *R. e. caldwelli* and *R. episcopus* ssp.) consistently exceeded 3.0%.

### 3.3. Mitogenomic Phylogeny and TMRCA

The newly assembled complete mitochondrial genomes of *Rhinolophus* sp., *R. osgoodi*, and *R. e. caldwelli* ranged in length from 16,848 to 16,871 bp (Table S5). These slight length variations were primarily attributed to indels within the non-coding regions, particularly the D-loop region. All mitogenomes exhibited a conserved structural organization and a strong AT bias, with AT content ranging from 56.2% to 56.6%.

**Figure 6:**
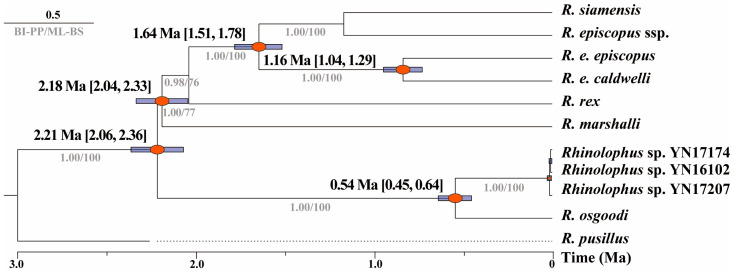
Mitogenomic chronogram showing phylogenetic relationships and divergence times within the “*R. macrotis* group,” based on 13 concatenated protein-coding genes. The blue bars at nodes represent the 95% highest posterior density (HPD) intervals for divergence time estimations. Numbers below branches indicate Bayesian posterior probabilities (BI-PP) and maximum likelihood bootstrap support (ML-BS) values, respectively. *Rhinolophus pusillus* was used as the outgroup. Time scale is in million years ago (Ma).

By integrating newly generated mitogenomic sequences with published datasets, we successfully assembled the 13 concatenated PCGs for all recognized taxa within the “*R. macrotis* group.” Phylogenetic reconstructions based on both the complete mitochondrial genomes and the 13 PCGs yielded highly congruent mitochondrial gene trees (Figure 6 and Figure S3). These analyses nested *Rhinolophus* sp. within the “*R. macrotis* group,” recovering it as the sister lineage to *R. osgoodi* with high support (BI-PP = 1.00; ML-BS = 100%).

TMRCA estimates revealed that the “*R*. *macrotis* group” originated approximately 2.21 Ma (95% HPD: 2.06–2.36 Ma; Figure 5). Subsequently, *R. marshalli* and *R. rex* diverged sequentially from the ancestral node. Further along the phylogeny, *R. siamensis* was recovered as nested within the three subspecies of *R. episcopus*, with their coalescent time estimated at 1.64 Ma (95% HPD: 1.51–1.78 Ma). Most recently, *Rhinolophus* sp. and *R. osgoodi* formed a single clade, sharing a most recent common ancestor dating to 0.54 Ma (95% HPD: 0.45–0.64 Ma).

## 4. Discussion

### 4.1. Integrative Taxonomy Reveals a Candidate Lineage

In this study, we conducted an integrative taxonomic assessment of *Rhinolophus* sp. by systematically comparing its phenotypic and genetic disparities with closely related taxa. Morphologically, these taxa exhibit high similarity in both external and craniodental linear parameters; however, they display profound divergence in their echolocation resting frequencies (Figure 3 and Figure 4). Furthermore, genetic markers with different inheritance modes yielded incongruent phylogenetic patterns and delimitation results (Figure 5), underscoring the complex evolutionary history of this species group. Among all examined close relatives, *Rhinolophus* sp. shares the highest phenotypic and mitochondrial affinities with *R. osgoodi*. Nevertheless, there is a significant divergence in their echolocation resting frequencies.

As long-distance signals, acoustic traits frequently diverge prior to other phenotypic characteristics [62,63]. For nocturnal insectivorous bats, echolocation is essential for spatial orientation, foraging, and intraspecific communication [64,65,66]. The echolocation resting frequency (RF) of the constant frequency component has been associated with *Rhinolophus* speciation [67] and is mostly species-specific in rhinolophid and hipposiderid bats [68,69], and is thus frequently utilized as a diagnostic acoustic feature for species delimitation [70,71]. Consistent with this, we observed marked RF divergence among these closely related taxa with no interspecific overlap, despite the presence of intraspecific variation (Figure 4F). Notably, the maximum intraspecific acoustic variation within *R. osgoodi* and *R. e. episcopus* can reach up to approximately 5 kHz (Table S1). Previous studies have demonstrated that such intraspecific acoustic variation can be influenced by environmental factors, including ambient noise, humidity, and temperature [72,73,74]. Furthermore, physical attributes such as age, sex, body size, and overall body condition can also induce shifts in the resting frequency [75,76]. Here, our relatively small sample size (*n* = 3) precludes a comprehensive assessment of intraspecific RF variation within *Rhinolophus* sp. However, the RF of *Rhinolophus* sp. is at least 10 kHz lower than the recorded frequency of *R. osgoodi* (Table S1), the magnitude of which substantially exceeds the maximum observed intraspecific variation within *R. osgoodi*. More importantly, when closely related bat species occur sympatrically, they frequently experience intense selective pressures to partition dietary resources and avoid acoustic interference [77,78,79]. The pronounced divergence in echolocation frequency observed between *Rhinolophus* sp. and *R. osgoodi* potentially reflects ecological character displacement [80,81], indicating ecological and behavioral differentiation. Nevertheless, because we currently lack detailed data on their specific roosting microhabitats, foraging environments, and body temperatures, we cannot entirely rule out the potential influence of these ecological and physiological factors on their acoustic frequencies.

For horseshoe bats, the complex noseleaf functions as a dynamic beamforming structure, undergoing active shape changes during pulse emission to precisely modulate and direct echolocation signals [82,83]. Therefore, the observed morphological modification in the noseleaf is likely functionally coupled with the significant shift in resting frequency. To facilitate comparative analyses with historical morphometric data, our study evaluated key morphological parameters of the noseleaf based on traditional linear measurements. Although morphological variations in the lancet and secondary leaflet were visually observed in the field, the obtained linear metrics failed to discriminate *Rhinolophus* sp. and *R. osgoodi* (Figure 3 and Figure 4). Given that noseleaf characters have been shown to be effectively used as traits for the identification of potential cryptic species of Rhinolophidae [84] and are crucial diagnostic traits for delimiting these taxa, future taxonomic evaluations should incorporate geometric morphometrics to finely analyze the three-dimensional structure of the noseleaf and precisely quantify its shape variation [85].

Similarly, cranial structures associated with the nasal cavity (e.g., nasal chamber size) are functionally linked to echolocation emission [86]. However, comparisons of craniodental linear measurements did not reveal discernible differences among these taxa (Figure 3 and Figure 4), highlighting the inherent limitations of standard linear measurements in capturing complex structural shifts. Future research could employ geometric morphometrics or micro-CT scanning for a more fine-scaled quantification and comparison of craniodental architectures [70,87]. More importantly, due to the rarity of this cryptic lineage, our analyses of external and craniodental morphology were restricted to a very limited sample size, making it difficult to entirely rule out the confounding effects of intraspecific variation or aberrant individuals. Under conditions of such low sample sizes, utilizing dimensionality reduction techniques like principal component analysis and Gaussian mixture models to infer morphological clustering can potentially yield misleading results [88]. Therefore, the interpretation of our current morphological findings warrants strict caution.

Genetic evidence provides additional context for the evolutionary status of *Rhinolophus* sp. Earlier studies suggested that general mammalian intraspecific mitochondrial divergence typically falls below 2%, and in most cases, is less than 1% [89]. A recent comprehensive analysis of mammalian phylogenies proposed a minimum K2P threshold of 1.5% for species separation, noting that divergence values between 1.5% and 2.5% represent an ambiguous range where intraspecific variation often exceeds predictions, thereby necessitating multi-locus validation [90]. The sequence divergence between *Rhinolophus* sp. and *R. osgoodi* is 1.4–2.0% (Table 1), falling squarely into this ambiguous range. Although this divergence is comparable to the established interspecific divergence between recognized species within this group, such as *R. osgoodi* and *R. e. episcopus* (1.7–2.3%), it cannot definitively distinguish between intraspecific variation and genuine interspecific divergence. Furthermore, we incorporated additional genetic information from two nuclear introns, *Chd1* and *Acox2*, which have been successfully applied for species delimitation in the *R. macrotis* complex [33,34]. However, in this study, the nuclear phylogeny revealed a topological discordance with the mitochondrial tree, clustering *Rhinolophus* sp. with *R. episcopus* and *R. siamensis*, albeit with low statistical support (Figure 5).

Additionally, in contrast to the mitochondrial *Cytb* gene, the species delimitation based on nuclear markers yielded overly conservative results (Figure 5). As a purely distance-based approach, ABGD does not rely on an underlying genealogical tree and can theoretically be applied to nuclear sequences that have undergone recombination [91]. However, this method is highly sensitive and often performs poorly in systems characterized by extremely small sample sizes per species (e.g., *n* < 3–5) or shallow genetic divergence [91,92]. Similarly, although we incorporated a tree-based method (i.e., mPTP), it exhibited extreme instability across our datasets and tended toward severe over-lumping in nuclear data. Such biases are likely driven by specific population traits, complex demographic processes, or uneven sampling of the species [57,93]. Consequently, the algorithmic species delimitation results presented herein should be interpreted as preliminary hypotheses. Comprehensive multi-locus coalescent frameworks, such as BPP or SNAPP, can be applied to genome-scale data to fully resolve the species boundaries within this group.

Therefore, integrating morphological, acoustic, genetic and ecological evidence, we conservatively designate *Rhinolophus* sp. as a candidate lineage within the “*R. macrotis* group.” However, given the observed mito-nuclear discordance and the complex evolutionary history characterizing this group [33,34], a formal taxonomic revision and species description require a more comprehensive assessment.

### 4.2. Mito-Nuclear Discordance Between Rhinolophus sp. and R. osgoodi

Discordance across independent datasets is a pervasive phenomenon in recently diverged lineages, as documented in diverse taxa such as tree frogs (genus *Boophis*) [22], lizards (genus *Sceloporus*) [23], the *Sicista subtilis* complex [24], and bats within the subfamily Vespertilioninae [94]. Consistent with previous studies [32,33], we observed profound mito-nuclear discordances within the “*R. macrotis* group.” Several evolutionary processes, including natural selection, sex-biased dispersal, historical introgression, and incomplete lineage sorting (ILS), are frequently invoked to explain such discordance [8]. These mechanisms likely account for the specific pattern observed between *Rhinolophus* sp. and *R. osgoodi*, wherein *Rhinolophus* sp. exhibits distinct nuclear phylogenetic affinities despite retaining a mitochondrial genome closely nested within *R. osgoodi*.

First, although mitochondrial genes were traditionally considered neutral markers, numerous studies have demonstrated that they can be subject to natural selection [95]. For instance, signals of positive selection have been detected in two mitochondrial genes encoding proteins of the oxidative phosphorylation system within the genus *Rhinolophus* [96]. Therefore, the observed mitochondrial affinities in our study might reflect convergent evolution driven by similar selective pressures. Second, sex-biased dispersal in temperate bats is predominantly male-mediated, which typically results in low levels of nuclear genetic differentiation among populations [97]. This pattern directly contradicts our observations of distinct divergence in nuclear phylogeny (Figure 5). Third, the current sympatric distribution of these two lineages strongly implicates historical or ongoing secondary contact [98,99]. Such contact frequently facilitates asymmetrical mitochondrial introgression following initial divergence, a phenomenon previously documented not only between *R. osgoodi* and *R. episcopus* [33] but also across various other horseshoe bats [100,101].

Alternatively, due to the short divergence time, ancestral mitochondrial polymorphisms may not have had sufficient time to achieve reciprocal monophyly, resulting in ILS [102,103]. Typically, given their smaller effective population sizes, high mutation rates, and lack of recombination, mitochondrial genes achieve coalescence more rapidly than nuclear genes [104], thereby exhibiting higher differentiation among closely related species. However, this theoretical expectation contrasts with the observed high mitochondrial genetic similarity (Figure 5 and Figure 6). Although the phylogenetic reconstruction based on *Chd1* and *Acox2* failed to recover *Rhinolophus* sp. and *R. osgoodi* as monophyletic clades, the species delimitation based on the nuclear dataset clustered them into a single putative species. This pattern reflects either a high degree of nuclear sequence similarity or insufficient genetic information to support robust species boundaries. Consequently, this preliminary nuclear dataset failed to definitively determine whether selection, introgression, or ILS primarily drives the observed mito-nuclear discordance.

Furthermore, our TMRCA estimation reveals that the diversification of the “*R. macrotis* group” initiated approximately 2.21 Ma (Figure 6), indicating a relatively recent divergence. This timeline coincides with the early Pleistocene, a period characterized by frequent global climatic shifts [105]. However, these divergence times should be interpreted with caution, as they were estimated solely based on a fixed and generalized mitochondrial substitution rate without fossil calibrations. Interestingly, our inferred timeline aligns closely with Chornelia et al. (2022), who, using fossil-calibrated *COI* markers, estimated the ancestral divergence of the *R. macrotis*, *R. marshalli*, and *R. rex* clade at approximately 3.22 Ma [106]. Conversely, studies employing multi-locus nuclear data have proposed a substantially older evolutionary origin for the “*R. macrotis* group” during the Late Miocene [27,101], a period characterized by sustained global cooling.

This temporal discrepancy highlights a potential bias in TMRCA estimations. Specifically, the relatively younger divergence times inferred from our mitochondrial dataset may be underestimated due to several compounding factors. First, our estimates are fundamentally derived from a mitochondrial gene tree rather than a true species tree; topological discordances between gene trees and species trees can inherently confound divergence time calculations [107,108]. Second, the mutational saturation of mitochondrial DNA can obscure ancient divergences, although this phenomenon is typically more pronounced over deeper evolutionary timescales [109,110]. More importantly, mitochondrial introgression among ingroup lineages can also lead to a substantial underestimation of the actual speciation times [111]. Therefore, future phylogenomic studies incorporating genome-wide nuclear markers and robust fossil calibrations are required to establish a more accurate and comprehensive evolutionary timeline for this group.

With the advent of high-throughput sequencing, genomic data have provided a powerful new tool for species delimitation and contributed to the widespread application of modern integrative taxonomy [112]. Recent studies of cryptic bat complexes increasingly rely on genome-scale datasets to resolve shallow divergences and mito-nuclear discordance [113,114,115,116]. Across Asia, particularly within its recognized biodiversity hotspots, systematic surveys have revealed extraordinary cryptic diversity, including horseshoe bats [117,118,119,120]. However, taxonomic evaluations for many of these regional taxa still predominantly rely on single-locus mitochondrial genes and traditional phenotypic data, frequently lacking comprehensive assessments of nuclear genomic variations. Although the present study integrated markers with different inheritance modes, the two selected nuclear introns lacked sufficient phylogenetic signal. This limitation, compounded by the restricted sample size of *Rhinolophus* sp., precluded a definitive taxonomic classification. Nevertheless, by applying an integrative taxonomic framework, our findings established a promising preliminary hypothesis of lineage divergence. Advancing the application of modern integrative taxonomy within Asian biodiversity hotspots is crucial for establishing a robust and definitive taxonomic foundation for future biodiversity conservation and evolutionary research.

## 5. Conclusions

By integrating morphological, acoustic, and multi-locus genetic data, we revealed pronounced phenotype-genotype and mito-nuclear discordances between *Rhinolophus* sp. and its closely related congeners, indicating a complex evolutionary history. Specifically, while it shares broad morphological similarity and highly nested mitochondrial affinities with *R. osgoodi*, it remains distinguishable by its divergent echolocation resting frequency. Furthermore, species delimitation revealed overly conservative nuclear signals, with nuclear phylogenies positioning *Rhinolophus* sp. closer to two other species, *R. episcopus* and *R. siamensis*. Given the currently limited sample size, which precludes a robust assessment of intraspecific variation, we provisionally designate *Rhinolophus* sp. as a candidate lineage within the “*R. macrotis* group.” Future integration of broad-scale population sampling, whole-genome sequencing, and geometric morphometrics will be essential to fully elucidate the taxonomy and evolutionary trajectory of this group. Finally, this study highlights the indispensable utility of an integrative framework in the delimitation of phenotypically conservative and recently diverged lineages, contributing to the ongoing reassessment of hidden chiropteran diversity within Asian biodiversity hotspots.

## Figures and Tables

**Table 1:** Pairwise K2P genetic distances among *Rhinolophus* sp. and its allied taxa based on the mitochondrial *Cytb* gene.

Species/Subspecies	*Rhinolophus* sp. (*n* = 3)	*R. osgoodi* (*n* = 41)	*R. e. episcopus* (*n* = 49)	*R. e. caldwelli* (*n* = 5)	*R. episcopus* ssp. (*n* = 13)	*R. siamensis* (*n* = 30)	*R. rex* (*n* = 4)	*R. marshalli* (*n* = 4)
*Rhinolophus* sp.	-	0.003	0.004	0.006	0.007	0.006	0.007	0.008
*R. osgoodi*	0.017	-	0.003	0.004	0.005	0.005	0.005	0.007
*R. e. episcopus*	0.026	0.020	-	0.003	0.005	0.005	0.005	0.006
*R. e. caldwelli*	0.036	0.025	0.019	-	0.005	0.005	0.005	0.007
*R. episcopus* ssp.	0.041	0.035	0.032	0.027	-	0.004	0.006	0.007
*R. siamensis*	0.043	0.036	0.033	0.027	0.017	-	0.006	0.008
*R. rex*	0.048	0.039	0.036	0.033	0.043	0.045	-	0.007
*R. marshalli*	0.056	0.051	0.048	0.050	0.053	0.057	0.052	-

Values below the diagonal represent the mean genetic distances, while values above the diagonal indicate the standard errors. Numbers in parentheses denote the sample sizes for each taxon. The hyphen (-) indicates not applicable.

## Data Availability

Data are contained within the article and Supplementary Materials. The data presented in this study are available upon request from the corresponding authors.

## Supplementary Materials

The following supporting information can be downloaded at: https://www.mdpi.com/article/10.3390/biology15110846/s1, Figure S1: Principal component analysis (PCA) scatter plots of *Rhinolophus* sp. and its allied taxa along the first and third principal components (PC1 vs. PC3); Figure S2: Bayesian Information Criterion (BIC) plots for Gaussian mixture model (GMM) clustering; Figure S3: Mitogenomic phylogenetic relationships among *Rhinolophus* sp. and closely related taxa; Table S1: Sample information, external morphological measurements, echolocation resting frequencies, and mclust assignments of the *Rhinolophus* specimens used in this study; Table S2: Craniodental measurements and mclust assignments of the *Rhinolophus* specimens used in this study; Table S3: Primer sequences used for the amplification of the mitochondrial genome; Table S4: GenBank accession numbers for the mitochondrial and nuclear gene sequences used in this study; Table S5: Nucleotide composition and basic characteristics of the complete mitochondrial genomes of *Rhinolophus* sp. and its closely related taxa; Table S6. PCA factor loadings, eigenvalues, and variance explained for external and craniodental datasets; Table S7: Alignment characteristics and optimal evolutionary models for the mitochondrial and nuclear datasets.
