# Supplementary material for: Integrative Taxonomy Reveals a Candidate Lineage Within the Rhinolophus macrotis Group
Source: Biology (Basel). 2026 May 28;15(11):846. doi: 10.3390/biology15110846 (PMC13255616; doi:10.3390/biology15110846)
Supplement: Supplementary file 1 [file biology-15-00846-s001.zip › Supplementary Figures.pdf]

Supplementary Figure

Integrative Taxonomy Reveals a Novel Cryptic Lineage within the *Rhinolophus macrotis* Group

Jinhua Cong [1,2,†,] Jiajun Zhang [1,2,†,] Haoran Yu [1,2,] Jinhong Lei [1,2,] Guiyin Miao [1,2,] Heran Yang [1,2,] Qiuchen Li [1,2,] Zhejia Zhang [1,2,] Gábor Csorba [3,] Keping Sun [4,*,] and Tong Liu [1,2,*]

[1] Jilin Provincial International Cooperation Key Laboratory for Biological Control of Agricultural Pests, Jilin Agricultural University, Changchun, 130118, China

[2] Jilin Provincial Key Laboratory of Animal Resource and Ecological Security, Jilin Agricultural University, Changchun, 130118, China

[3] Department of Zoology, Hungarian Natural History Museum, 1088, Budapest, Hungary

[4] Key Laboratory of Vegetation Ecology of Education Ministry, Institute of Grassland Science, Northeast Normal University, Changchun 130024, China

[*] Correspondence: liut035@nenu.edu.cn (T.L.); sunkp129@nenu.edu.cn (K.S.)

[†] These authors contributed equally to this work.

* Correspondence: liut035@nenu.edu.cn (T.L.)

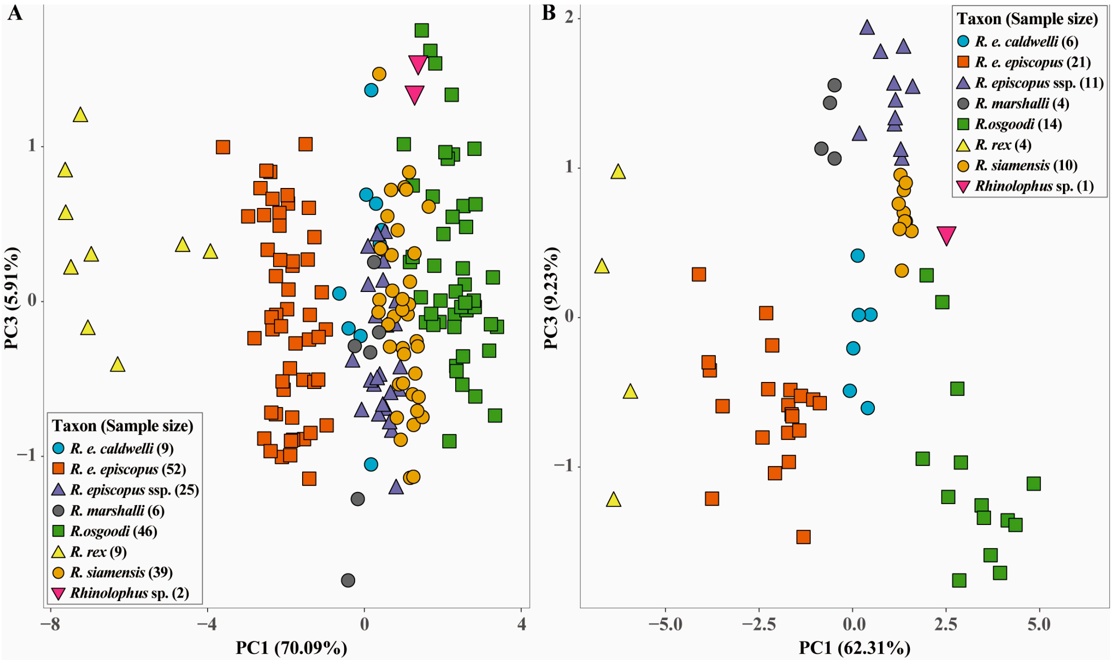

Figure S1. Principal component analysis (PCA) scatter plots of *Rhinolophus* sp. and its allied taxa along the first and third principal components (PC1 vs. PC3). Analyses are based on (A) seven external morphological parameters and (B) 11 craniodental morphological parameters. Different colors and geometric shapes denote a priori species or subspecies identifications. Numbers in parentheses within the legends indicate the corresponding sample sizes for each taxon.

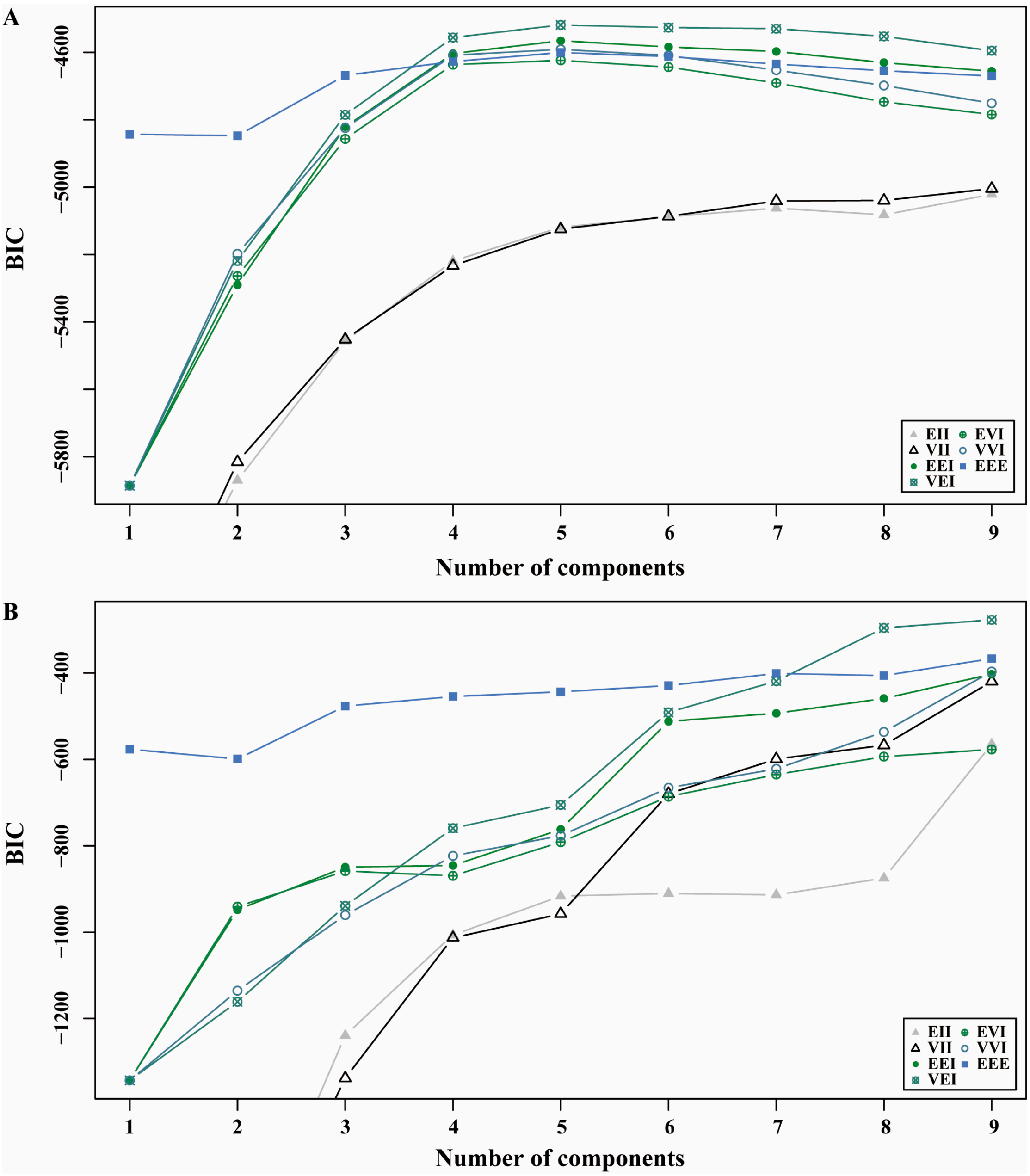

Figure S2. Bayesian Information Criterion (BIC) plots for Gaussian mixture model (GMM) clustering. The plots illustrate the BIC values across a range of predefined component numbers based on (A) external morphological parameters and (B) craniodental morphological parameters. Distinct lines and symbols denote different parameterizations of the covariance matrix. The optimal number of clusters and the best-fitting covariance structure are determined by the maximum BIC value.

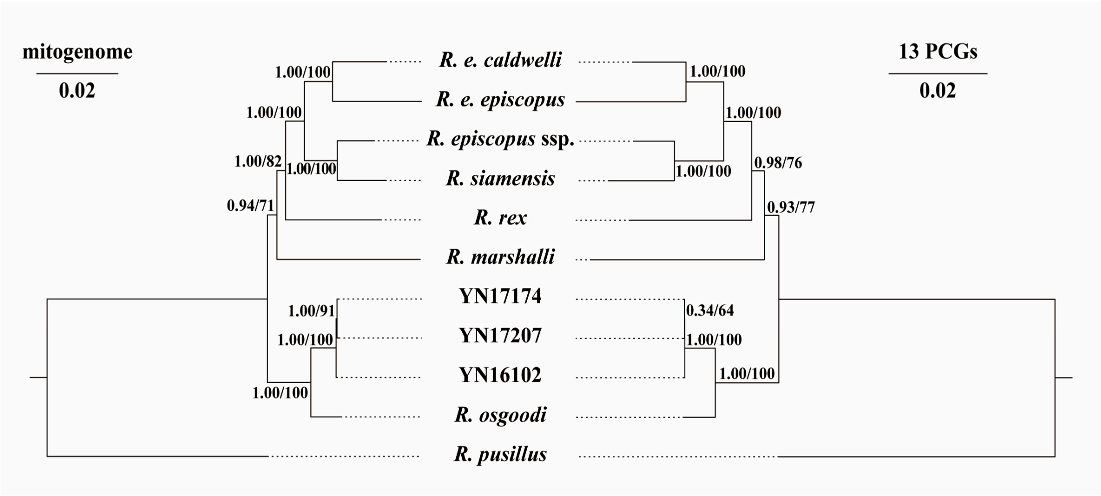

Figure S3. Mitogenomic phylogenetic relationships among *Rhinolophus* sp. and closely related taxa. The mirrored phylogenies are based on the complete mitochondrial genome (left) and the concatenated 13 protein-coding genes (PCGs) dataset (right). Numbers at the nodes indicate Bayesian posterior probabilities (BI-PP) and Maximum Likelihood bootstrap values (ML-BS), respectively. The scale bar represents 0.02 nucleotide substitutions per site.
